# Unrevealing the Potential of *Sansevieria trifasciata* Prain Fraction for the Treatment of Androgenetic Alopecia by Inhibiting Androgen Receptors Based on LC-MS/MS Analysis, and In-Silico Studies

**DOI:** 10.3390/molecules27144358

**Published:** 2022-07-07

**Authors:** Henny Kasmawati, Resmi Mustarichie, Eli Halimah, Ruslin Ruslin, Arfan Arfan, Nurramadhani A. Sida

**Affiliations:** 1 Doctoral Program in Pharmacy, Faculty of Pharmacy, Universitas Padjadjaran, Bandung 45363, Indonesia; 2 Faculty of Pharmacy, Universitas Halu Oleo, Kendari 93232, Indonesia; mahaleo241@yahoo.co.id (R.R.); arfan09@uho.ac.id (A.A.); apt.nurramadhani08@gmail.com (N.A.S.); 3 Department of Analytical Pharmacy and Medicinal Chemistry, Faculty of Pharmacy, Universitas Padjadjaran, Bandung 45363, Indonesia; 4 Department of Pharmacology and Clinical Pharmacy, Faculty of Pharmacy, Universitas Padjadjaran, Bandung 45363, Indonesia; eli.halimah@unpad.ac.id

**Keywords:** AGA, androgen receptor, dynamics simulation, molecular docking, *Sansevieria trifasciata* Prain

## Abstract

Androgenetic Alopecia (AGA) occurs due to over-response to androgens causing severe hair loss on the scalp, and requires the development of new and efficient drugs to treat this condition. This study explores and identifies secondary metabolites from *Sansevieria* *trifasciata* Prain using the LC-MS/MS and in-silico method. The inhibitory activity of bioactive compounds from *S. trifasciata* Prain against androgen receptors (PDB ID: 4K7A) was evaluated molecularly using docking and dynamics studies by comparing their binding energies, interactions, and stability with minoxidil. The results of the LC-MS/MS analysis identified Methyl pyrophaeophorbide A (**1**), Oliveramine (**2**), (2S)-3′, 4′-Methylenedioxy-5, 7-dimethoxyflavane (**3**), 1-Acetyl-β-carboline (**4**), Digiprolactone (**5**), Trichosanic acid (**6**) and Methyl gallate (**7**) from the leaves subfraction of this plant. Three alkaloid compounds (compounds **1**, **3**, and **4**), and one flavonoid (compound **2**), had lower docking scores of −7.0, −5.8, −5.2, and −6.3 kcal/mol, respectively. The prediction of binding energy using the MM-PBSA approach ensured that the potency of the four compounds was better than minoxidil, with energies of −66.13, −59.36, −40.39, and −40.25 kJ/mol for compounds **1**, **3**, **2**, and **4**, respectively. The dynamics simulation shows the stability of compound 1 based on the trajectory analysis for the 100 ns simulation. This research succeeded in identifying the compound and assessing the anti-alopecia activity of *Sansevieria trifasciata* Prain. Seven compounds were identified as new compounds never reported in *Sansevieria trifasciata* Prain. Four compounds were predicted to have better anti-alopecia activity than minoxidil in inhibiting androgen receptors through an in silico approach.

## 1. Introduction

Alopecia is currently one of the diseases of global concern. This disease is a dermatological disorder characterized by abnormal hair loss [1]. Based on research, about 60 to 70% of the world population suffers from androgenetic alopecia (AGA) caused by excess production of androgen 5α-dihydrotestosterone (5α-DHT) in hair follicles, especially in dermal papilla cells (DP) as a regulator of hair growth [2,3]. The 5α-DHT has a binding affinity that is five times higher than testosterone (T) against androgen receptors (AR). In addition, 5α-DHT also induces androgen-sensitive genes ten times higher than T. These two factors (AR and androgen-sensitive genes) are overproduced during AGA [4,5,6].

In addition to AR, 5α-reductase (5α-R) is also responsible for the occurrence of AGA [7]. 5α-R has two isoforms, 5α-R1 and 5α-R2, distributed in the liver, brain, epididymis, prostate, and hair follicles [8]. 5α-R becomes a more difficult target to achieve by drugs and has a critical role in prostate function [9]. This is indicated by the lack of effectiveness of finasteride and shows side effects such as abnormal ejaculation and sexual function, impotence, and gynecomastia [10].

One of the best possibilities for treating AGA is by inhibiting AR on the dermal papilla cells [11]. AR is highly expressed on the hair epithelium, beard, and dermal papilla cells, causing a decrease in the function of hair growth factors so that hair becomes miniature and falls out over time [12]. AR consists of three functional domains: (1) a ligand-binding domain (T and 5α-DHT ligands) at the C-terminal, (2) a DNA-binding domain (DBD) to regulate transcription, and (3) an N-terminal domain that functions as transactivation at the N-terminus [13,14]. When bound to AR, T or 5α-DHT ligands will be downregulated androgen-sensitive genes in DP cells and cause hair loss. Therefore, another way to reduce the effect of androgens in AGA hair loss is to inhibit the interaction between androgens and their receptors [14,15].

Currently, only two synthetic drugs have received FDA approval for treating AGA: minoxidil and finasteride [16,17]. Both drugs have side effects and are effective in <50% of patients. The anti-alopecia activity of minoxidil accelerates the telogen-exogen and anagen phase, shortening the telogen phase and increasing hair follicle size [18,19,20,21,22]. In contrast, finasteride can inhibit the activity of 5α-reductase type II from converting testosterone to DHT, which triggers alopecia [23,24]. The efficacy of these two drugs is less than optimal in reducing progressive hair loss and stimulating hair growth. After one year and four months of topical 5% minoxidil administration, studies have shown that only about 38.6% of the subjects showed hair growth progression [25,26,27]. Scalp irritation that can occur due to topical minoxidil, in addition to suboptimal efficacy, are several reasons for patients to search for new alternative treatments using traditional plants [20,21,28,29]. Therefore, searching for and exploring new drugs to treat AGA is necessary.

The richness of compounds in natural sources provides an excellent opportunity for drug discovery and development, especially as a guide for modern drugs [30]. Natural compounds have unique chemical structures and various pharmacological properties [31]. One of the plants known to be traditionally used to treat alopecia is *Sansevieria trifasciata* Prain [32]. This plant has several bioactive compounds such as alkaloids, flavonoids, steroids, terpenoids, and tannins [33,34]. In previous studies, linoleic acid in *Sansevieria trifasciata* Prain can interact with 5α-reductase receptors to prevent alopecia by prolonging the anagen phase of hair growth [10,35]. However, there are still minimal studies of the constituents of this plant on AR.

Based on these descriptions, we are interested in investigating the anti-alopecia activity of the leaves of *Sansevieria trifasciata* Prain. Furthermore, the bioactive compounds in this plant were identified through LC-MS/MS analysis, followed by the in-silico method using docking and dynamic studies toward the androgen receptor. This study is expected to provide scientific information as an alternative for developing new drugs that are safe and effective for the treatment of AGA, which is sourced from natural products.

## 2. Results

### 2.1. Phytochemical Constituent by LC-MS/MS Analysis

Our previous study of the anti-alopecia activity in vivo assay showed that *Sansevieria trifasciata* Prain is a potential candidate to be developed as an anti-alopecia herbal. Four active subfractions were obtained: subfractions C, D, E, and F [36]. In this study. The compound of each subfraction was analyzed using the LC-MS/MS method (Supplementary Materials). This method has better selectivity, sensitivity, and accuracy for fast analysis [37,38]. According to LC-MS/MS analysis results, subfractions C, D, E, and F obtained eighteen compounds. Furthermore, the MS database identified nine probable compounds: alkaloids such as 1-Acetyl-β-carboline, methyl pyrophaeophorbide A and oliveramine, flavonoids such as (2S)-3′, 4′-methylenedioxy-5, 7-dimethoxyflavane, monoterpenes digiprolactone, phenolic methyl gallate, and fatty acid trichosanic acid (Table 1). These compounds have never been reported in *Sansevieria trifasciata* Prain.

### 2.2. Molecular Docking Simulation

The inhibitory activity of the compounds identified from the LC-MS/MS method of the *Sansevieria trifasciata* Prain subfractions against androgen receptors was estimated using a proper docking procedure. The root mean square deviation (RMSD) criteria become a reference in ensuring the rationality of the procedure. The rationality of the docking procedure can be determined using the Root Mean Square Deviation (RMSD) criterion of the minoxidil heavy atoms between the redocked poses and the experimental poses (crystallography). The quality of the binding poses search was suitable when the RMSD value was less than 2.0 Å [39,40]. As a native ligand, minoxidil was redocked to the androgen receptor, resulting in a conformational shift of 1.64 Å from its crystallographic x-ray position. These results illustrate the ability of the docking protocol to predict the best conformation of bioactive compounds in this plant subfraction. All compounds were docked to the binding site of minoxidil on the androgen surface, as shown in Table 2.

Table 2 shows that five bioactive compounds in *Sansevieria trifasciata* Prain have a lower docking score than native ligands and other compounds. Interestingly, all the compounds identified have different structures. Compound **1** had the lowest docking score with −7.0 kcal/mol energy, followed by compound 2 with a binding energy of −6.3 kcal/mol. Compounds 3 and 4 also gave satisfactory results with relative differences in binding energies, namely −0.6 kcal/mol with −5.8 and −5.2 kcal/mol. Uniquely, minoxidil displays the same predicted binding energy as compound 6 at −4.2 kcal/mol. Compared to the native ligand, there is a slight difference in compound 5 with −4.5 kcal/mol and compound 7 with −4.0 kcal/mol.

**Figure 1:**
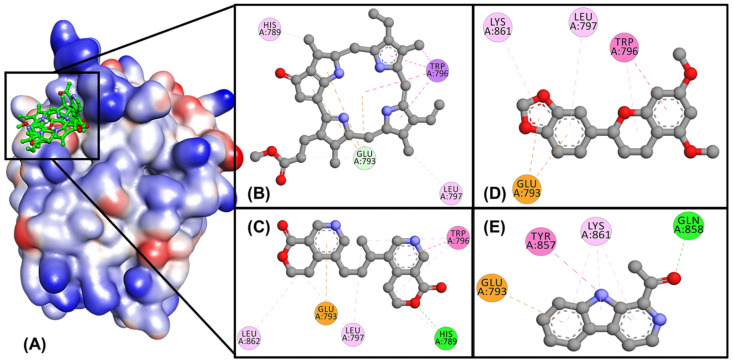
(**A**) Molecular interactions of all compounds in active site AR. 2D interactions of (**B**) compound 1 (Methyl pyropheophorbide A), (**C**) compound 2 (Oliveramine), (**D**) compound 3 ((2S)-3′, 4′-Methylenedioxy-5, 7-dimethoxyflavone), and (**E**) compound 4 (1-Acetyl-β-carboline) with AR.

Molecular interaction analysis of the results of the docking study revealed that all compounds were able to bind to the cofactor binding site except compound 7 (Figure 1A). This catalytic site was in the residue region of TYR857, GLN858, LYS861, GLU793, TRP796, and LEU797. We suspect that this could be one of the reasons the binding energy of compound 7 is lower than that of other compounds. Here, we described the best four compound interactions based on docking scores and compared them with minoxidil. The interaction of compound 1 shows that the carboxyl-methyl group forms a hydrogen bond (H-bond) to GLU793 with a distance of 3.76 Å. Hydrophobic interactions with TRP796, LEU797, and HIS789 in each methyl-cyclopentane group were also observed (Figure 1B). In compound 2, there are two different H-bonds, namely residues HIS789 and LEU862, with oxygen atoms in each tetrahydro-pyran ring (Figure 1C). These two H-bonds have a distance of 2.15 Å and 4.98 Å, respectively. The similarity of hydrophobic interactions like compound **1** also appears in this compound.

In comparison, the interaction of minoxidil with AR shows the presence of two hydrogen carbon bonds in the pyrimidine group at residues GLU793 (3.47 Å) and TRP796 (3.88 Å), and hydrophobic interaction with the LYS861 on the piperidine ring, which stabilizes the ligand binding to the receptor [22]. In contrast to the other compounds, compound **3** did not show the presence of an H-bond with the AR receptors but had three hydrophobic interactions with residues TRP796, LEU797, and LYS861 (Figure 1D). Uniquely, compound **4** forms an H-bond with a GLN858 residue on its carbonyl group, which is not observed in the other compounds with a distance of 2.0 Å, and hydrophobic interaction with the TYR857 residue (Figure 1E). Finally, all compounds formed Pi-Anion bonds with the GLU793 residue at the AR catalytic site.

In Compound **5**, this compound’s hydroxyl and carbonyl groups were observed to form two hydrogen bonds to the residues GLU793 and LYS861 with a distance of 2.72 Å and 3.70 Å, respectively. This compound also observed a hydrophobic interaction with the TYR857 residue in the methyl group. Compounds **6** and **7** form different hydrogen bonds with other compounds. In compound **6**, hydrogen bonds are created on the ARG854 residue of the carbonyl at a distance of 2.04 Å. This compound showed a hydrophobic interaction with the LEU797 residue at the methyl chain’s end. In compound **7**, the hydroxy group displays hydrogen bonds with two residues, SER853 (2.83 Å) and ARG855 (2.46 Å), while simultaneously forming hydrophobic interactions with the same residue.

### 2.3. Molecular Dynamics Simulation

**Figure 2:**
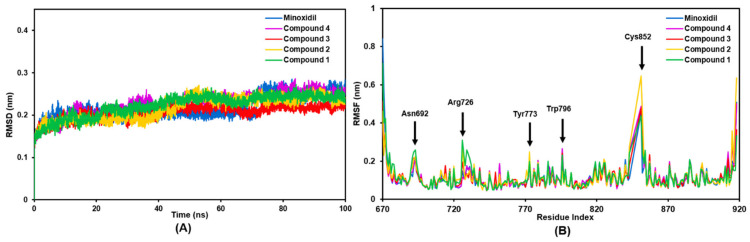
The plots of (**A**) RMSD of backbone atoms for AR-compound complex, and (**B**) RMSF of backbone atoms for AR-compound complex during 100 ns of simulation.

The best four compounds from the docking stage were proposed to predict their dynamic behavior to AR over 100 ns through MD simulation. The stability and flexibility assessment of the complex was analyzed based on the RMSD and RMSF criteria. The RMSD can be seen in Figure 2A, where all compounds were relatively stable during the 100 ns simulation with complex fluctuations below 0.3 nm. Minoxidil and the test compounds showed a similar fluctuation pattern. They did not differ significantly, with an average RMSD of 0.217 nm. Compounds **1** and **4** had the highest average fluctuations recorded at 0.223 and 0.226 nm. Minoxidil as a comparison and compound **2** had a similar average fluctuation slightly lower than the previous two compounds with a value of 0.216 nm. Interestingly, the RMSD pattern with the average fluctuation was given by compound **3** with a value of 0.206 nm. This stable pattern indicates that both the test and comparison ligand have the ability to stabilize the complex during the simulation.

**Figure 3:**
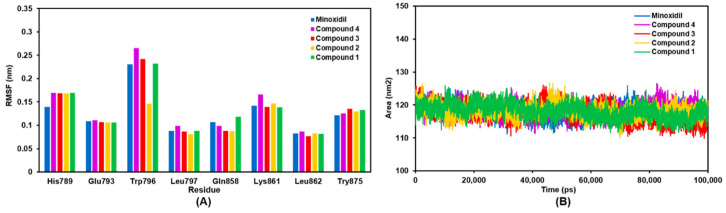
The plots of (**A**) RMSF of AR catalytic site AR, and (**B**) SASA area for AR-compound complex during 100 ns of simulation.

Analysis of the RMSF residue numbers in the AR backbone region showed that all complexes had similar oscillations (Figure 2B). The RMSF values were high in some residues, such as ASN692, ARG726, TYR773, and TRP796, with fluctuations of ~0.23 nm. The CYS852 residue in the loop region of AR displays a very high peak intensity in compound 2 with a fluctuation value of ~0.6 nm and other compounds at ~0.45 nm. Meanwhile, the GLN670 and THR918 residues are AR’s N-terminus and C-terminus regions, producing the highest fluctuations. Specifically, the researchers were interested in characterizing the intensity of amino acid fluctuations on the AR catalytic site (Figure 3A). This plot illustrates a similar pattern of residual fluctuations in all compounds. The highest fluctuation at this site was found in the residue TRP796 with a value of ~0.25 nm. Interestingly, these residues were only compound 2 of moderate-intensity with a value of ~0.15 nm.

Solvent-accessible surface area (SASA) analysis was performed on each complex to complete the stability analysis (Figure 3B). An SASA plot displays the predicted areas on the receptor accessible to water molecules during the simulation. The smaller the area accessed by the water molecule, the more stable the ligand-receptor complex. The mean score of the SASA analysis was also calculated. The minoxidil-AR, **1**-AR, **2**-AR, and **4**-AR complexes had the same area accessible by water molecules of 118 nm^2^. The smallest area is found in the **3**-AR complex, with an access area of 117 nm^2^. This result aligns with the RMSD pattern, which shows that compound **3** has better stability than the other compounds.

**Figure 4:**
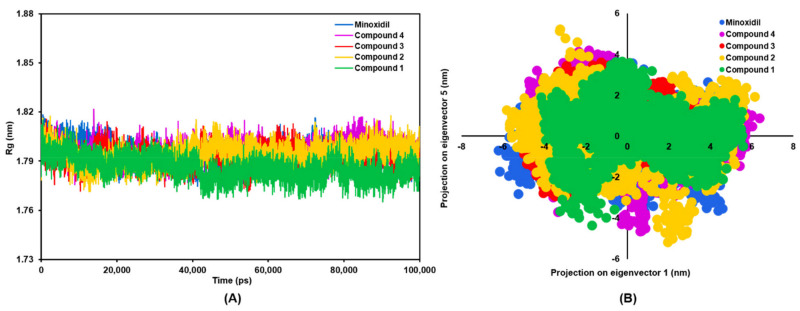
The plots of (**A**) radius of gyration of backbone atoms for AR-compound complex, and (**B**) principal component analysis of the projected trajectory in 2D, during 100 ns of simulation.

Measurement of the radius of gyration (Rg) was carried out to assess the compactness of the receptor during the simulation (Figure 4A). A low Rg value indicates a stable level of receptor folding. On the other hand, if the protein is in the unfolded form, the Rg value will vary during the simulation. It can be seen in the graph that the minoxidil-AR, **2**-AR, **3**-AR, and **4**-AR complexes have similar compactness from the beginning to the end of the simulation, with an average Rg value of 1.794 nm. The results showed that the **1**-AR complex had the lowest Rg value of 1.785 nm, which indicated that this complex had better compactness than other compounds.

The researchers identified and analyzed the overall essential dynamics pattern of the AR-ligand complex using principal component analysis (PCA). Most of the protein fluctuations can be explained by the low and high projections on the eigenvectors. The motion of the backbone atoms is captured in these two eigenvectors and visualized on a 2D trajectory plot (Figure 4B). Suppose the motion of the backbone atoms during the simulation in each AR-ligand complex is similar. In that case, the eigenvectors and eigenvalues should be similar. The stable AR-ligand complex can be identified from the less space occupied by the cluster during the simulation. On the other hand, varied clusters that occupy more space show less stable complexes. On 2D eigenvector plots, complex **1**-AR was found to occupy less space than AR-minoxidil and other complexes. However, the vector patterns formed from all complexes tend to be similar.

### 2.4. Binding Energy Calculation of the Complexes

This research analyzes the binding energy with the MM-PBSA approach to compare the affinity of each compound against AR. Several energies that affect binding affinity are examined, including Van der Waals (∆E_VDW_), electrostatic (∆E_Ele_), polar solvation (∆E_PS_), and SASA energy (∆E_SASA_). All these energies are calculated in kJ/mol (Table 3). All compounds were predicted to have lower binding energies (∆E_Bind_) than minoxidil (−34.64 kJ/mol). Compound **1** has the most substantial binding energy with a −66.13 kJ/mol value, followed by compound **3** with an energy of −59.36 kJ/mol. These results are suitable with predictions based on RMSD, Rg, SASA, and PCA complexes which revealed that these two compounds were more stable than the other compounds.

Compounds **2** and **4** had almost the same binding energies with −40.39 kJ/mol and −40.25 kJ/mol, respectively. These compounds were present in subfraction C, which showed a stronger affinity for AR than minoxidil. The analysis results show that the Van der Waals energy has the most significant effect on binding to AR. Electrostatic energy and SASA contributed negative energy, but it was not substantial. Meanwhile, the polar solvation energy is less favorable for the AR complex.

### 2.5. Pharmacokinetic and Toxicity Prediction

The four selected compounds were then predicted for their pharmacokinetic profiles (Table 4). All compounds had good permeability to the skin (log Kp < −2.5). Compounds with good skin permeability (SP) can increase the potential activity of the compound when administered topically. A total of three compounds were predicted not to break the blood-brain barrier (BBB). However, only compound **4** was expected to be able to pass BBB (log BBB > 0.3). It was observed that compounds **3** and **4** had central nervous system (CNS) values > −2, indicating that these compounds can penetrate the CNS. All compounds are predicted not to inhibit the metabolism of drugs or cholesterol, thus showing a good safety profile. The potential for mutagenicity and hepatotoxicity, respectively, could be caused by compounds **2** and **4** and compounds **1** and **2**. Finally, all compounds showed good safety in the absence of compounds predicted to cause skin sensitivity. The skin permeability and sensitivity profile provide a potential perspective of this plant because alopecia treatment is mainly applied topically to the skin.

## 3. Discussion

We obtained as many as nine main subfractions (subfraction A-F) from the fractionation process for methanol extract of *Sansevieria trifasciata* Prain with different yield percentages. The low percentage yield (≤5%) was found in subfraction D (3% *w*/*w* yield). Subfractions A, E, and F have moderate yield percentages of 14%, 12%, and 11%, respectively. Finally, subfractions B and C produced the highest yields with percentages of 35% and 25%, respectively. Based on the previous identification, we decided to further analyze the C-F subfraction with LC-MS/MS. The analysis of four subfractions identified seven compounds (compounds **1**–**7**). Compounds **2** and **6** were found in the root of *Gentiana straminea* Maxim and Pomegranate Seed Oil (PSO). These two compounds were reported to have anti-inflammatory and antidiabetic activity in rats [41,42]. Compounds **5** and **4** of *Moringa oleifera* and marine *actinomycete* were reported to have antibacterial activity against MRSA bacteria [43,44].

Compound **7** was identified in this plant to have various activities, including antitumor activities by inhibiting tumor infiltration of CD4^+^ CD25^+^ regulatory, affected cell membrane integrity, causing a decrease in cytoplasmic pH, and membrane hyperpolarization of *Vibrio cholera*, and particular inhibitor of herpes simplex virus [45,46,47]. However, there is a lack of information about compounds **1** and **3** on their activity on *Sansevieria trifasciata* Prain or other sources. Information about compound **1**, which is known to have photodynamic activity [48]. Compounds **1**–**4** in this study were the first to be informed on the possibility present in *Sansevieria trifasciata* Prain based on analysis by LC-MS/MS. The minimal information about the potential of the seven bioactive compounds in alopecia activity prompted us to investigate them based on molecular docking and dynamics simulations of androgen receptors crystallized from human hair dermal papilla cells [22].

Androgen receptors are one of the essential targets that regulate hair growth factors (androgen-sensitive genes) [49]. In alopecia, DHT acts by binding to androgen receptors, causing a decrease in the function of hair growth factors so that hair becomes miniature and falls out over time [12]. We also investigated AR inhibition based on molecular docking and dynamics simulations to ascertain the potency of the bioactive compounds from *Sansevieria trifasciata* Prain as an alopecia drug candidate. It was observed that five bioactive compounds could bind to the AR catalytic site while having the highest affinity compared to minoxidil. Five compounds (**1**–**5**) had lower binding energies than minoxidil, with an energy range of −7.0 kcal/mol to −4.5 kcal/mol. One compound (**6**) was estimated to have a minoxidil equivalent binding energy of −4.2 kcal/mol, and one compound (**7**) was lower with energy of −4.0 kcal/mol. The docking study showed that all the best compounds interacted with catalytic residues such as Glu793, Trp796, and Lys861 in AR with an average distance of <4 Å. These three residues are responsible for hydrogen bond and hydrophobic interaction formation during AR activity. These three residues are responsible for creating hydrogen bonds and hydrophobic interactions during AR activity. The binding with this residue is an indication that the compounds **1**–**4** of *Sansevieria trifasciata* Prain can inhibit AR and have promising potential to treat alopecia.

Compounds **1**–**4** were continued to the dynamics simulation stage to evaluate their binding affinity, stability, and flexibility to AR. We observed that the binding trend obtained after MD simulation was very stable, with RMSD values below 0.3 nm. The effect of protein movement during the MD simulation can be observed from fluctuations in amino acids. The highest intensity fluctuations were seen in CYS852 in the loop region and GLN670 and THR918 in the N-terminus and C-terminus regions. All compounds showed stable conformational based on complex stability analysis (sasa area, radius of gyration and principal component analysis). Compounds **1**–**4** provide the lowest predictive binding energy than minoxidil. These four compounds show good safety without causing skin irritation. The permeability and sensitivity profile of the skin provides a potential perspective of this plant because the treatment of alopecia is mainly applied topically to the skin.

## 4. Materials and Methods

### 4.1. Extraction and Separation Compounds

The *Sansevieria trifasciata* Prain leaves were collected from the Kambu District, Kendari City, Southeast Sulawesi Province. The leaves were prepared by harvesting the samples, wet sorting, washing with water, deforming the shape by cutting the sample into small pieces, and then drying in an oven (Air Performance Ovens Frailabo^®^) at 50 °C to obtain dry simplicia and ground into powder. The simplicia powder (3500 g) was macerated with ethanol as a solvent and evaporated into a crude extract (467.46 g) with yield of 13.36% (*w*/*w*). The extract was then fractionated with n-hexane, ethyl acetate, and water. The ethyl acetate fraction was used for further separation with column chromatography. The ethyl acetate fraction was impregnated on 200–350 mesh silica gel which is two times the weight of the sample. Silica gel 60H_254_ p.a (E. Merck) weighed as much as 20–40 times the weight of the sample and was put into the column while compacting with a vacuum pump. The sample was then eluted with eluent (n-hexane: EtOAc: MeOH) in various gradients. The result of separation with column vacuum obtained sixteen subfractions, and subfractions with similar separation profiles on TLC were combined. Nine main subfractions were obtained: subfraction A (combined subfractions 1–4) (14% *w*/*w* yield), B (subfraction 5–7) (35% *w*/*w* yield), C (subfraction 8–9) (25% *w*/*w* yield), D (subfraction 10) (3% *w*/*w* yield), E (subfraction 11–14) (12% *w*/*w* yield) and F (subfraction 15–16) (11% *w*/*w* yield). The active subfractions C-F were used for further LC-MS/MS analysis.

### 4.2. LC-MS/MS Analysis

The chemical compound of subfraction C, D, E, and F was analyzed using LC-MS waters Xevo G2-XS Quadrupole time of flight mass spectrometry equipped with an electrospray ionization interface (ESI). The ESI source was performed in positive ion mode between *m/z* 50 and 1200 with optimization parameters: acquisition time 0–17 min, high CE ramp 10–40 eV, collision energy 6 eV, cone voltage 30 V, desolvation gas flow, and the temperature was set to 1000 L/h and 500 °C. The temperature of the column was set as 40 °C. Formic acid 0.1% in water (eluent A) and formic acid 0.1% in acetonitrile (eluent B) were used as a solvent with a flow rate of 0.3 mL/min. The eluent composition were as follows: 0–1 min 5% eluent B, 11–14 min 100% eluent B, 17 min 5% eluent B. The samples were injected into the column with a volume of 1 µL, respectively. The sample was post-processed with Waters UNIFI^®^ version 1.8 software and compared with Waters build-in library database from the instrument (Waters Corp. Milford, MA, USA).

### 4.3. Molecular Docking Simulation

The AR crystal structure complexed with minoxidil was downloaded from the PDB website with access code 4K7A (https://www.rcsb.org/), accessed on 5 April 2022. First, with the help of the AutoDock Tools program version 1.5.6 [50], the water molecules crystallized with the receptor were removed. The addition of a polar hydrogen atom and a Kollman charge was applied to the receptor. Lastly, the receptor was prepared in PDBQT format. The compounds detected in the LC-MS/MS results were then collected in three dimensions from the PubChem database (https://pubchem.ncbi.nlm.nih.gov/), accessed on 5 April 2022. All compounds were freed from restraint and assigned a gasteiger charge by the same program in the receptor preparation process. The docking process of the bioactive compounds of the *Sansevieria trifasciata* Prain subfraction to androgen receptors was carried out using Autodock Vina [51]. The procedure was validated by a redocking process based on the RMSD criteria. The docking area is set to 27 × 27 × 27 A with a center point of 6.529 × 4.864 × −4.729 A (coordinates x, y, and z, respectively). The best poses from the docking stage were analyzed with Discovery Studio Visualizer.

### 4.4. Molecular Dynamics Simulation

All conformations with the lowest energy in the docking results were forwarded to the dynamics simulation stage with the GROMACS 2016 version 6 software [52]. The simulation was run for 100 ns by applying the AMBER99SB-ILDN force field [53]. Ligands were parametrically measured using ACPYPE [54]. Electrostatic forces of complex systems utilized Ewald’s particle mesh method [55]. The solvation process used a TIP3P cube-shaped water model at a temperature of 310 K and was neutralized with Na^+^ and Cl^−^ ions. The stability of the complex was determined by analyzing the parameters root-mean-square deviation and fluctuation (RMSD and RMSF), solvent-accessible surface area (SASA), gyration radius (Rg), and 2D projection of principal component analysis (PCA). The binding energies of the AR-complex were also estimated using the MM/PBSA approach following the previous protocol [56], and their absorption, distribution, metabolism, excretion, and toxicity (ADMET) profile were determined by the pkCSM web server [57].

## 5. Conclusions

This research successfully identified potential compounds from the subfraction of *Sansevieria trifasciata* Prain leaves as anti-alopecia treatment in inhibiting androgen receptor activity based on LC-MS/MS analysis and computational studies. A total of seven identified bioactive compounds have never been reported in *Sansevieria trifasciata* Prain. The compounds Methyl pyrophaeophorbide A (**1**), Oliveramine (**2**), (2S)-3′, 4′-Methylenedioxy-5, 7-dimethoxyflavane (**3**), and 1-Acetyl-β-carboline (**4**) have anti-alopecia activity based on an in-silico study by inhibiting androgen receptors. Based on molecular docking scores and MM-PBSA, they showed a lower predictive value of binding energy than minoxidil. Furthermore, a dynamics study showed that the four compounds showed similar stability based on RMSD, RMSF, and SASA analysis. However, Methyl pyrophaeophorbide A (**1**) was more stable on Rg and PCA analysis during the 100 ns simulation. These four compounds have never been reported to have anti-alopecia activity through an in silico approach in inhibiting androgen receptors.

## Figures and Tables

**Table 1:** The *m/z* profile of identified compounds in subfractions C, D, E, F of *Sansevieria trifasciata* Prain.

Sample	Rt (Min)	Formula	Observed *m/z*	Neutral Mass (Da)	Identification
Subfraction C	5.33	C_13_H_10_N_2_O	211.0870	210.07931	1-Acetyl-ß-carboline
	8.82	C_20_H_20_N_2_O_4_	353.1467	352.14231	Oliveramine
	9.40	C_18_H_30_O_2_	279.2327	278.22458	Trichosanic acid
	10.01	-	313.1585	-	-
	10.19	C_35_H_36_N_4_O_5_	592.2685	593.27650	Candidate Mass C_35_H_36_N_4_O_5_
Subfraction D	5.81	C_18_H_18_O_5_	315.1232	314.11542	(2S)-3′, 4′-Methylenedioxy-5, 7-dimethoxyflavane
	5.81	C_31_H_27_N_3_O_15_	682.1497	681.14422	Candidate Mass C_31_H_27_N_3_O_15_
	10.19	C_35_H_36_N_4_O_5_	593.2766	592.26857	Candidate Mass C_35_H_36_N_4_O_5_
	10.98	C_37_H_40_N_4_O_6_	637.3024	36.294790	Candidate Mass C_37_H_40_N_4_O_6_
	11.57	C_35_H_38_N_4_O_3_	563.3035	562.29439	Candidate Mass C_35_H_38_N_4_O_3_
Subfraction E	5.80	C_18_H_18_O_5_	315.1230	314.11542	(2S)-3′, 4′-Methylenedioxy-5, 7-dimethoxyflavane
	10.63	C_36_H_38_N_4_O_7_	639.2824	638.27405	Candidate Mass C_36_H_38_N_4_O_7_
	10.98	C_36_H_38_N_4_O_5_	607.2924	606.28422	Candidate Mass C_36_H_38_N_4_O_5_
	11.31	C_34_H_36_N_4_O_3_	549.2870	548.27874	Methyl pyrophaeophorbide A
Subfraction F	3.37	C_8_H_8_O_5_	185.0438	184.03717	Methyl gallate
	3.63	C_11_H_16_O_3_	197.1165	196.10994	Digiprolactone
	9.37	C_18_H_30_O_2_	279.2321	278.22458	Trichosanic acid
	10.16	C_35_H_36_N_4_O_5_	593.2781	592.26857	Candidate Mass C_35_H_36_N_4_O_5_

**Table 2:** Docking scores of minoxidil and identified compounds in LC-MS with AR.

Identified Compounds	Compound’s Code	Docking Score (Kcal/mol)
Methyl pyrophaeophorbide A	**1**	−7.0
Oliveramine	**2**	−6.3
(2S)-3′, 4′-Methylenedioxy-5, 7-dimethoxyflavane	**3**	−5.8
1-Acetyl-β-carboline	**4**	−5.2
Digiprolactone	**5**	−4.5
Minoxidil	-	−4.2
Trichosanic acid	**6**	−4.2
Methyl gallate	**7**	−4.0

**Table 3:** MM/PBSA summary energy of minoxidil and the four best compounds against AR *.

Compounds	∆E_VDW_	∆E_Ele_	∆E_PS_	∆E_SASA_	∆E_Bind_
Minoxidil	−60.98	−12.99	47.05	−7.72	−34.64
**1**	−119.18	−12.96	78.56	−12.55	−66.13
**2**	−74.75	−10.16	52.65	−8.13	−40.39
**3**	−105.16	−3.30	60.46	−11.36	−59.36
**4**	−73.85	−9.14	50.73	−7.99	−40.25

* all values are in kJ/mol.

**Table 4:** ADMET Prediction Results.

Compounds	ADME					Toxicity		
	SP (logKP)	BBB (logBB)	CNS (logPS)	CYP2D6	TC (mL/min/kg)	AMES	HPT	SS
**1**	−2.854	0.038	−2.611	No	−0.411	No	Yes	No
**2**	−2.93	−0.651	−2.987	No	0.765	Yes	Yes	No
**3**	−2.787	−0.096	−1.647	No	0.214	No	No	No
**4**	−2.85	0.584	−1.39	No	0.481	Yes	No	No

## Data Availability

Data is contained within the article or Supplementary Materials.

## Supplementary Materials

The following supporting information can be downloaded at: https://www.mdpi.com/article/10.3390/molecules27144358/s1. Table S1. The *m/z* profile of identified compounds in subfractions C, D, E, F of *Sansevieria trifasciata* Prain.
