# Supplementary material for: Unrevealing the Potential of Sansevieria trifasciata Prain Fraction for the Treatment of Androgenetic Alopecia by Inhibiting Androgen Receptors Based on LC-MS/MS Analysis, and In-Silico Studies
Source: Molecules. 2022 Jul 7;27(14):4358. doi: 10.3390/molecules27144358 (PMC9318048; doi:10.3390/molecules27144358)
Supplement: Supplementary file 1 [file molecules-27-04358-s001.zip › molecules-1787896-supplementary.pdf]

Supporting information;

Unrevealing The Potential of *Sansevieria trifasciata* Prain Fraction For The Treatment of Androgenetic Alopecia by Inhibiting Androgen Receptors Based on LC-MS/MS Analysis, and in-silico Studies

Henny Kasmawati[1,4,*], Resmi Mustarichie[2,*], Eli Halimah[3], Ruslin Ruslin [4], Arfan Arfan [5], Nurramadhani A. Sida[6]

[1]Doctoral Program in Pharmacy, Faculty of Pharmacy, Universitas Padjadjaran, Bandung, Indonesia, 45363; hennykasmawati@uho.ac.id

[2]Department of Analytical Pharmacy and Medicinal Chemistry, Faculty of Pharmacy, Universitas Padjadjaran, Bandung, Indonesia, 45363; resmi.mustarichie@unpad.ac.id

[3]Department of Pharmacology and Clinical Pharmacy, Faculty of Pharmacy, Universitas Padjadjaran, Bandung, Indonesia, 45363; eli.halimah@unpad.ac.id

[4]Faculty of Pharmacy, Universitas Halu Oleo, Kendari, Indonesia, 93232; hennykasmawati@uho.ac.id (HK); mahaleo241@yahoo.co.id (RR); arfan09@uho.ac.id (AA); apt.nurramadhani08@gmail.com (NAS)

* Correspondence: hennykasmawati@uho.ac.id; (Henny Kasmawati), and resmi.mustarichie@unpad.ac.id (Resmi mustarichie)

Table S1 The m/z profile of identified compounds in subfractions C, D, E, F

1. The LC-MS/MS Analysis of FRACTION C sample

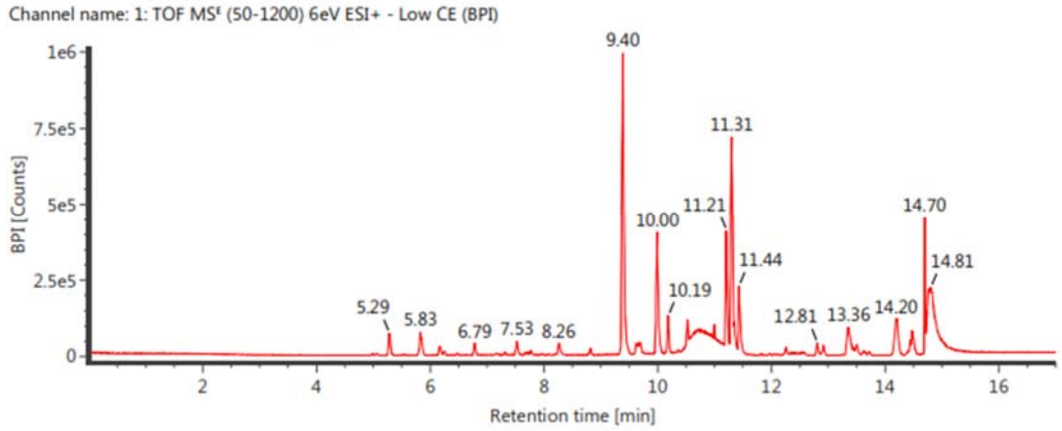

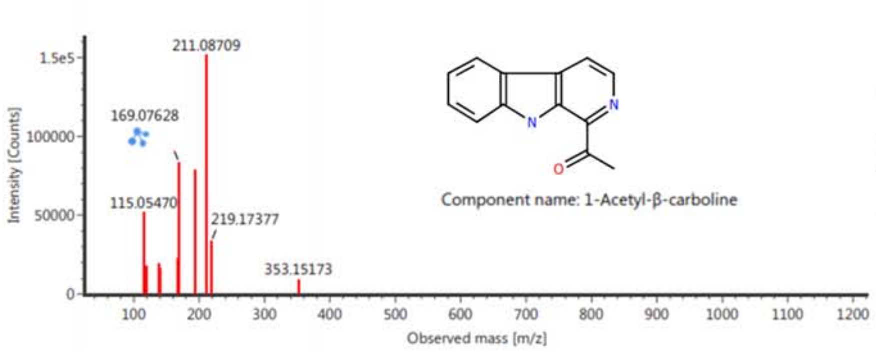

Peak 1

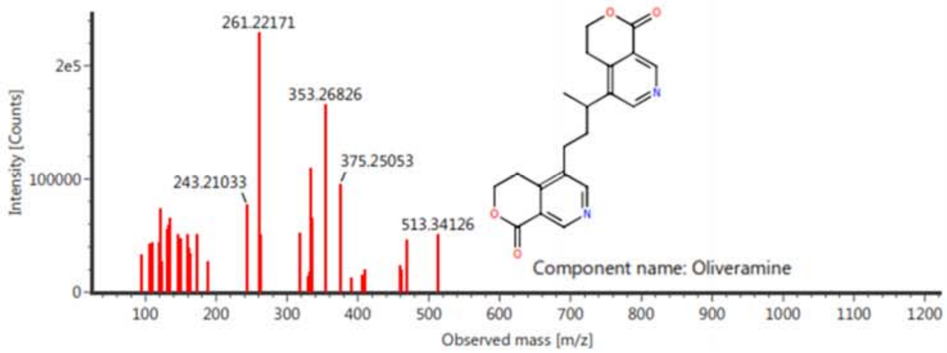

Peak 2

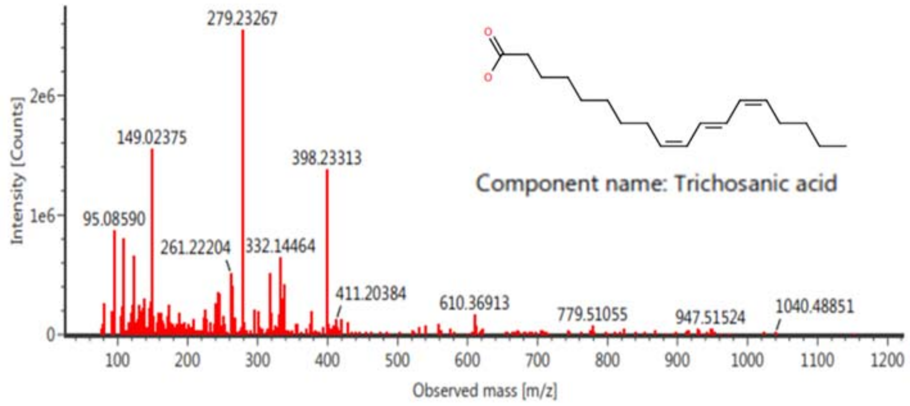

Peak 3

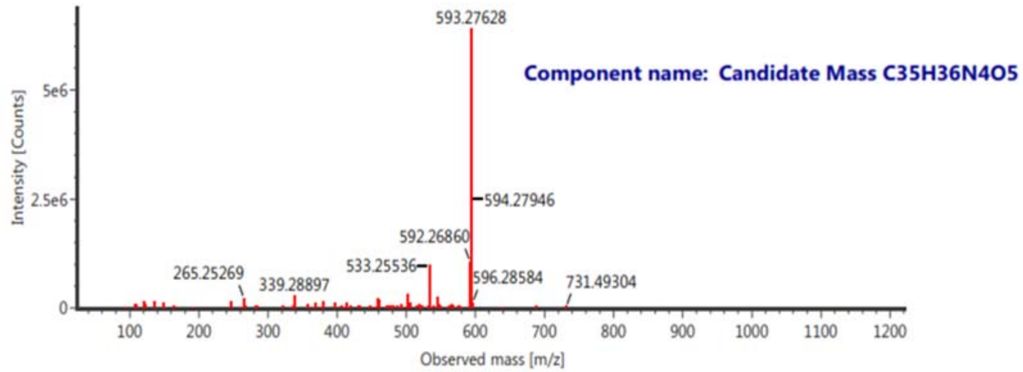

Peak 4

2. The LC-MS/MS Analysis of FRACTION D sample

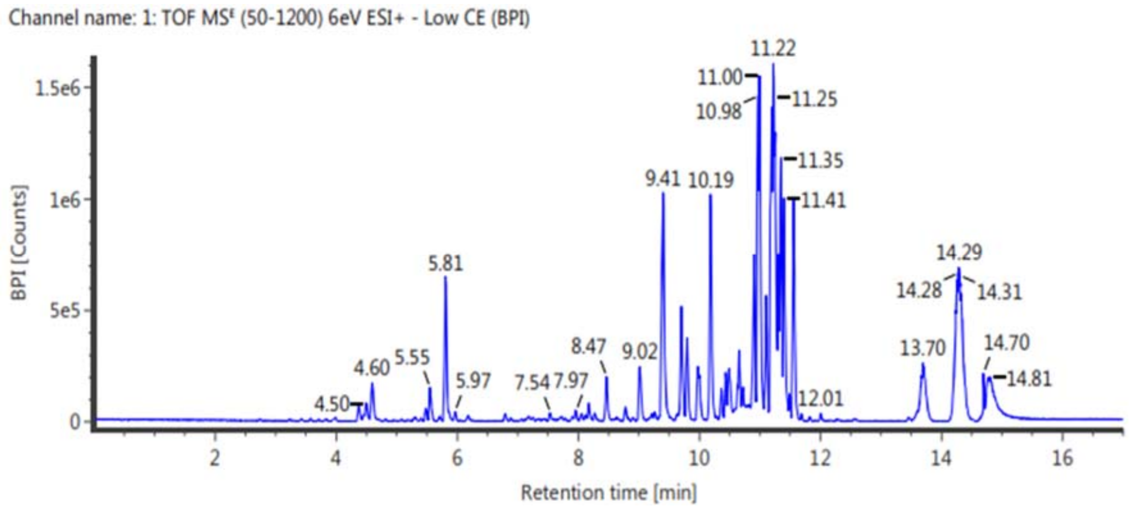

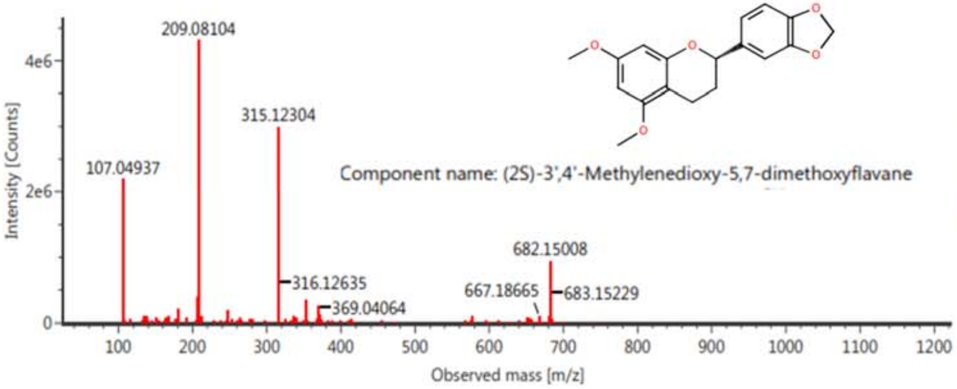

Peak 1

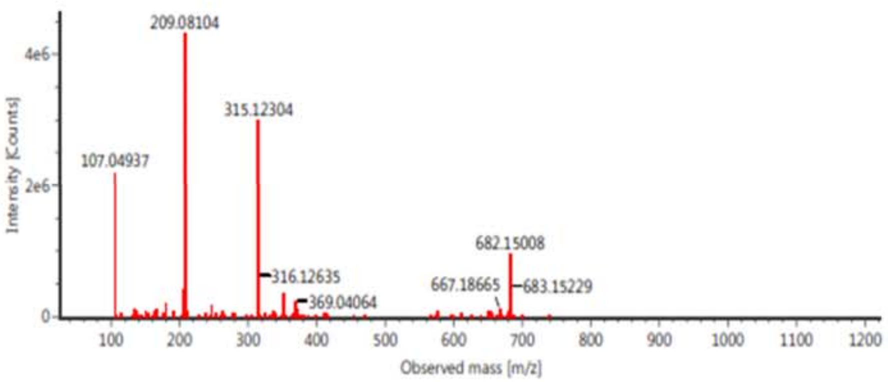

Peak 2

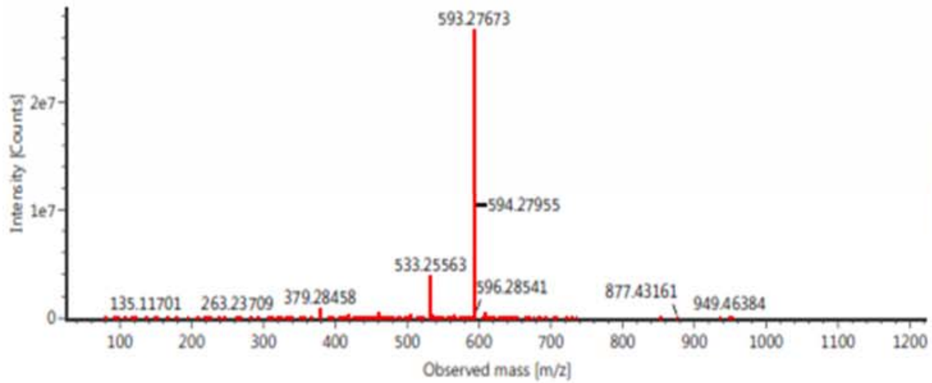

Peak 3

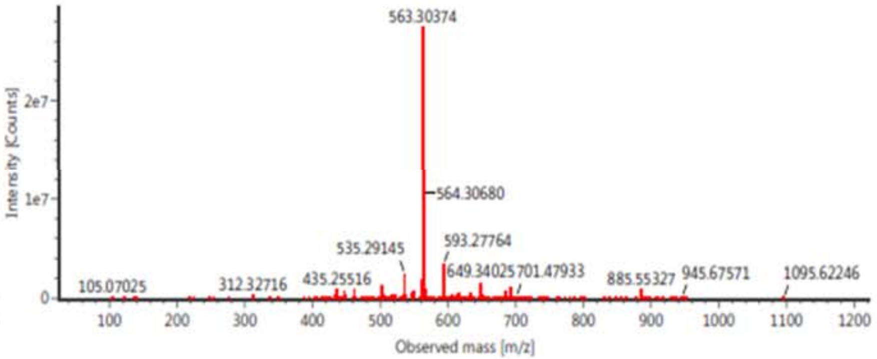

Peak 4

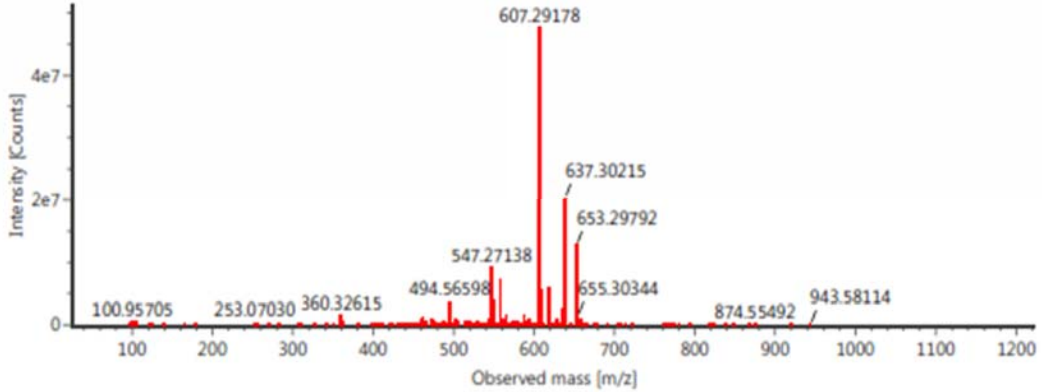

Peak 5

3. **The LC-MS/MS Analysis of FRACTION E sample**

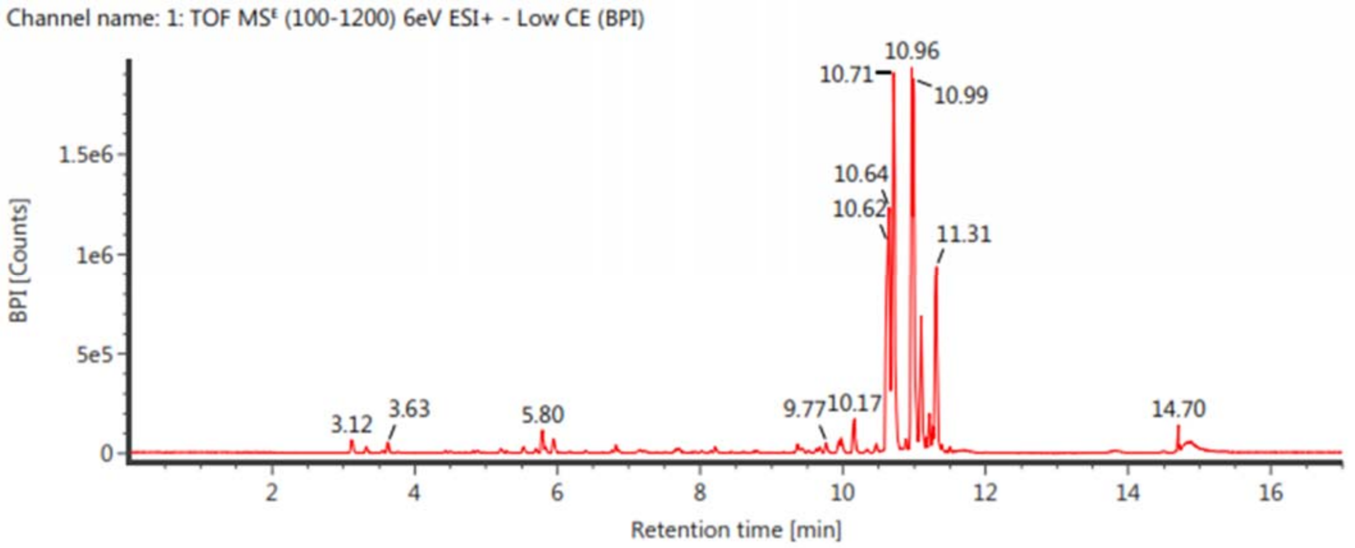

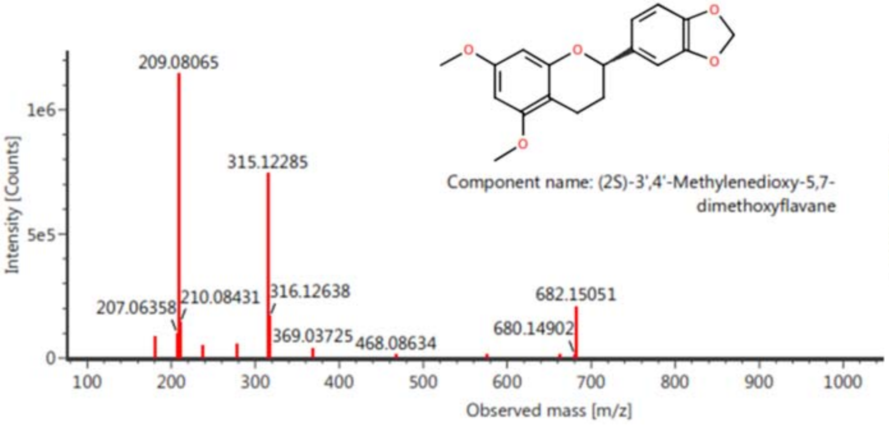

Peak 1

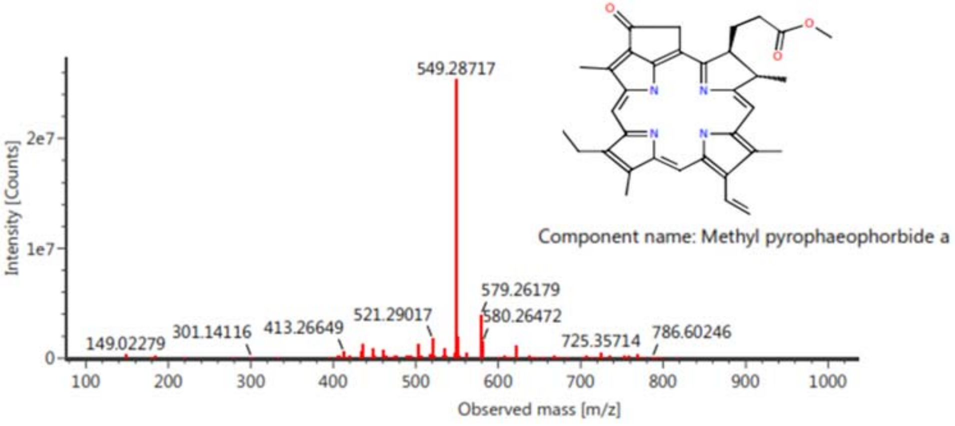

Peak 2

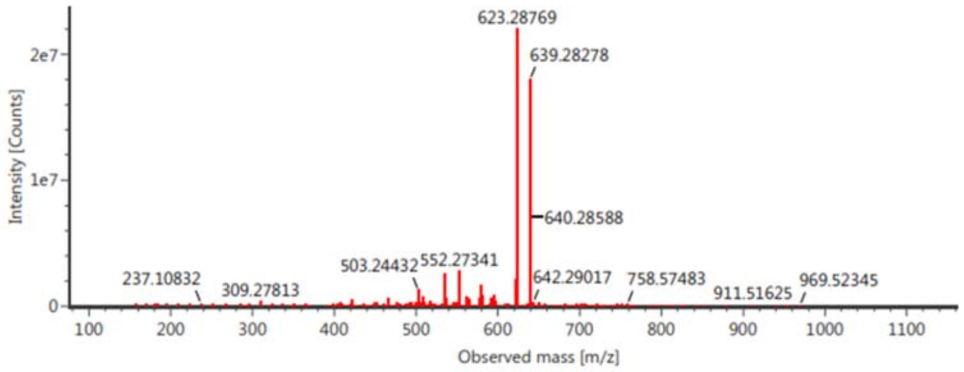

Peak 3

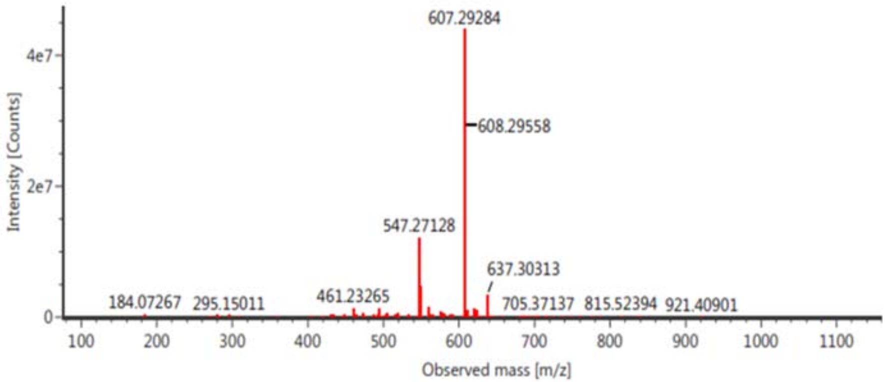

Peak 4

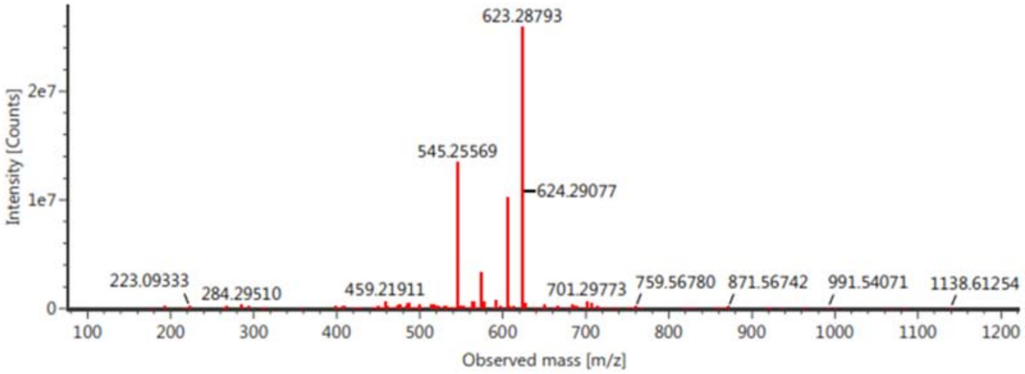

Peak 5

4. **The LC-MS/MS Analysis of FRACTION F sample**

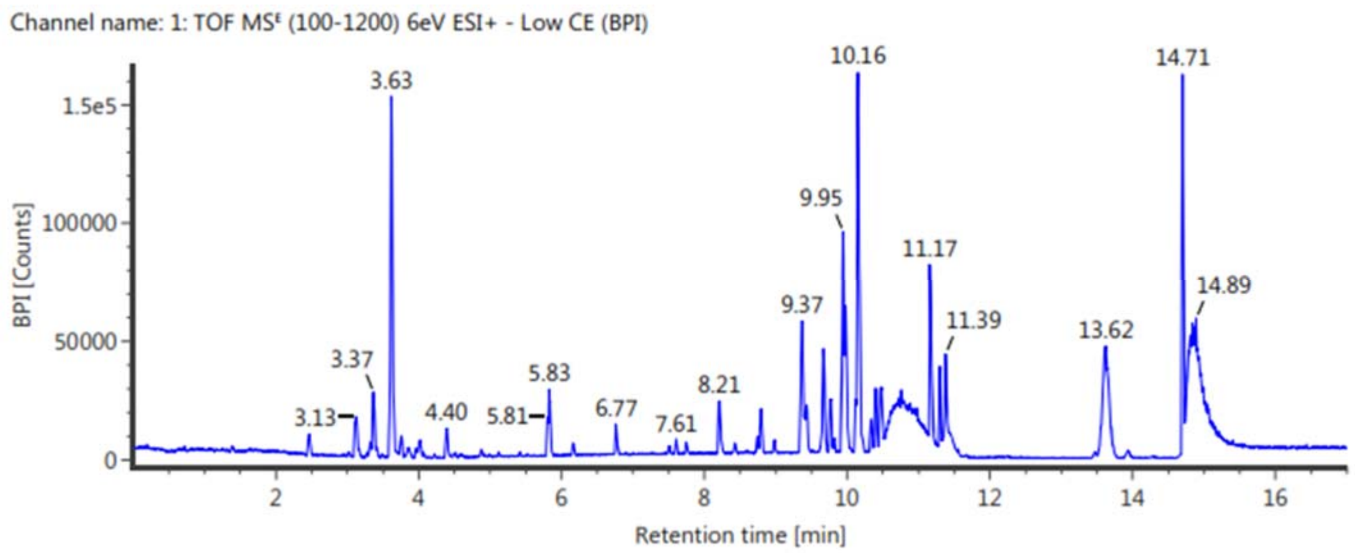

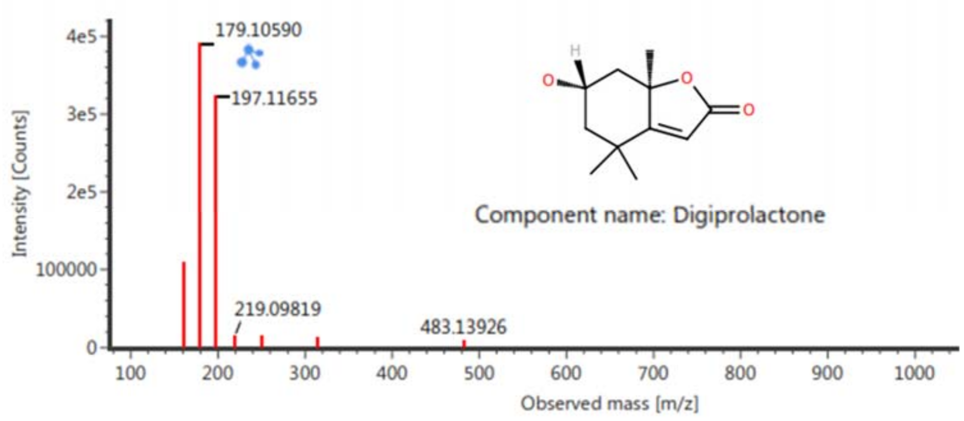

Peak 1

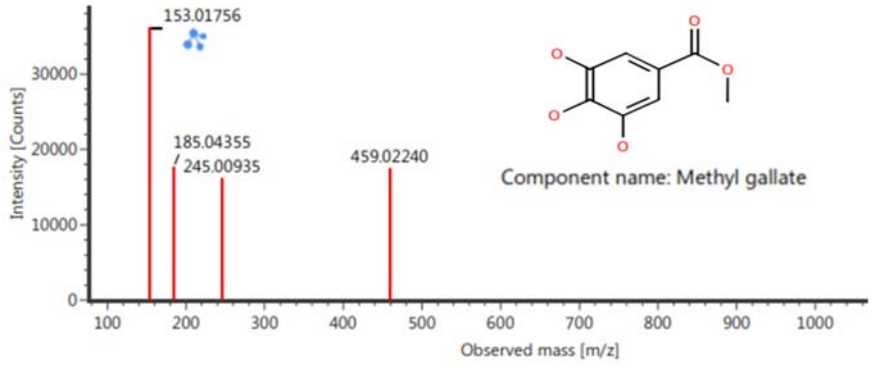

Peak 2

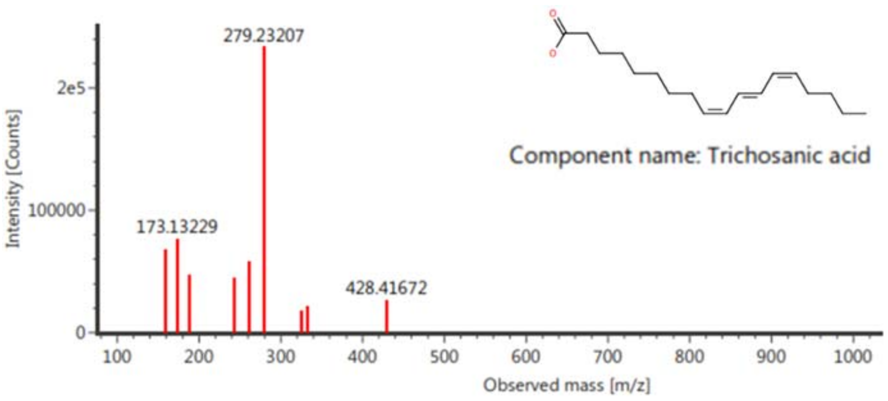

Peak 3

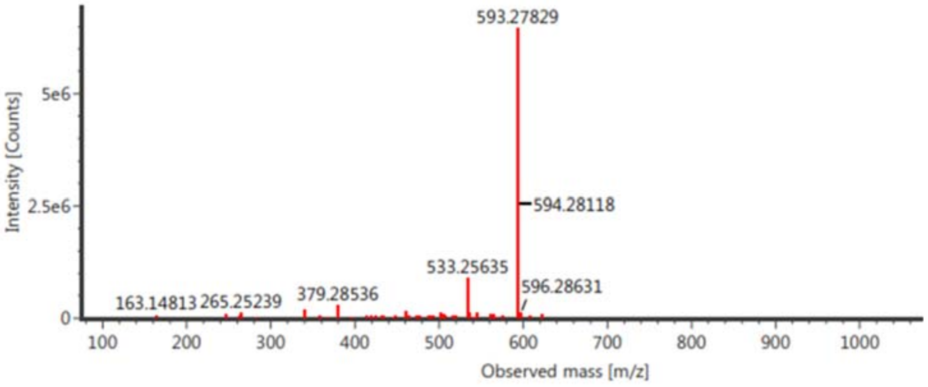

Peak 4

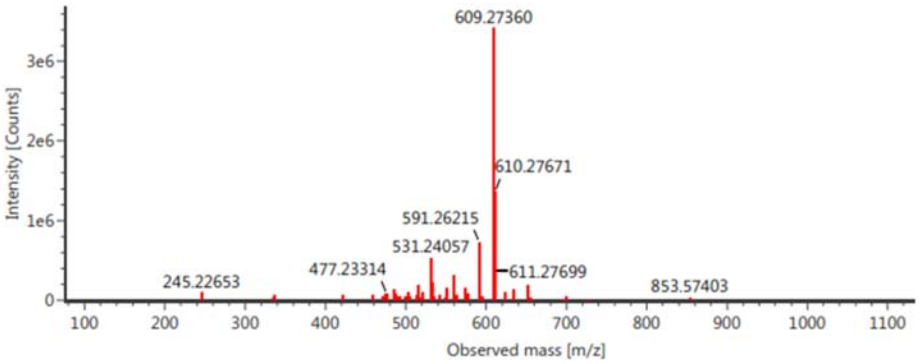

Peak 5
